# Techno-economic analysis of underground hydrogen storage in Europe

**DOI:** 10.1016/j.isci.2023.108771

**Published:** 2023-12-20

**Authors:** Mayukh Talukdar, Philipp Blum, Niklas Heinemann, Johannes Miocic

**Affiliations:** 1 Karlsruhe Institute of Technology (KIT), Institute of Applied Geosciences, Karlsruhe, Germany; 2 School of Geosciences, University of Edinburgh, Edinburgh, UK; 3 Energy and Sustainability Research Institute, University of Groningen, Groningen, the Netherlands

**Keywords:** Engineering, Energy Storage, Energy Modeling, Energy Management

## Abstract

Hydrogen storage is crucial to developing secure renewable energy systems to meet the European Union’s 2050 carbon neutrality objectives. However, a knowledge gap exists concerning the site-specific performance and economic viability of utilizing underground gas storage (UGS) sites for hydrogen storage in Europe. We compile information on European UGS sites to assess potential hydrogen storage capacity and evaluate the associated current and future costs. The total hydrogen storage potential in Europe is 349 TWh of working gas energy (WGE), with site-specific capital costs ranging from $10 million to $1 billion. Porous media and salt caverns, boasting a minimum storage capacity of 0.5 TWh WGE, exhibit levelized costs of $1.5 and $0.8 per kilogram of hydrogen, respectively. It is estimated that future levelized costs associated with hydrogen storage can potentially decrease to as low as $0.4 per kilogram after three experience cycles. Leveraging these techno-economic considerations, we identify suitable storage sites.

## Highlights

• Underground hydrogen storage potential in European natural gas sites is calculated
• Higher capital costs and lower levelized costs in salt cavern stores due to cyclicity
• Future levelized cost can decrease to 50% of current value in three experience cycles
• Storage site selection based on storage potential, capital, and levelized costs

## Graphical abstract

**Figure:**
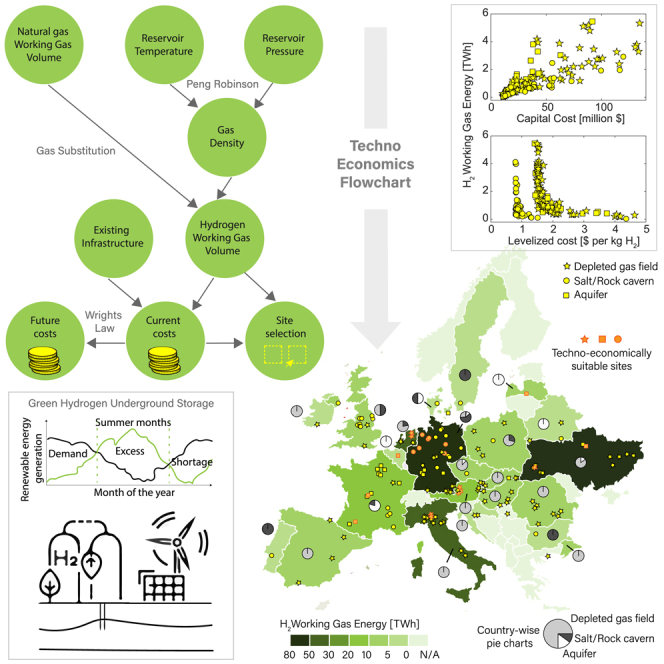

## Teaser

Earth sciences; Geology; Economic geology; Environmental science; Energy policyed costs

## Introduction

It is imperative to transition toward a climate-neutral society to meet the global climate targets outlined in the Paris Agreement and the European Union’s goal of achieving climate neutrality by 2050.^1^ Developing renewable energy systems that are affordable, reliable, and sustainable is a crucial step in this process and has recently received increased attention. To ensure system security and flexibility, the storage of excess renewable energy is an integral component of these energy systems. In high-latitude areas like Europe, renewable energy production varies with season.^2^ Long-term (monthly to seasonal) and large-scale (GWh-TWh) energy storage can therefore reduce seasonal supply and demand imbalances as part of the export supply chain.^3^ Currently, energy storage in Europe is dominated by subsurface hydrocarbon storage, particularly underground natural gas storage (UGS).^3^ Depleted gas fields, saline aquifers, and artificial rock caverns, often in salt rocks, are used for these subsurface storage operations.

Hydrogen, as a low-carbon energy carrier,^4^^,^^5^ has the potential to play a significant role as a fuel substitute for energy-intensive industries and can serve as an energy storage carrier by converting excess renewable energy into hydrogen via electrolysis and storing it for later use during periods of high energy demand.^6^ However, there is limited experience with subsurface storage of high-purity (>90%) hydrogen.^7^^,^^8^ The costs associated with subsurface hydrogen storage, which are dependent on the specific geological setting of the storage site, are not understood yet.^9^^,^^10^

As demand for hydrogen increases, repurposing depleted gas fields,^11^ aquifers, and salt caverns can provide a time-effective, cost-effective, and environmentally friendly solution for large hydrogen storage.^12^ This process requires modifications to the current infrastructure, including upgrades to compressors, wellheads, and pipelines, as well as modifications to the storage reservoirs to accommodate the different physical and chemical properties of hydrogen compared with natural gas.^13^^,^^14^

Site-specific studies on the feasibility of underground hydrogen storage have been performed in various locations.^15^^,^^16^^,^^17^^,^^18^ Regional studies have estimated hydrogen storage potential in salt deposits, depleted hydrocarbon reservoirs, aquifers, and underground gas storage sites or a combination of these.^19^ These regional estimation approaches and results differ from study to study, resulting in a wide range of reported hydrogen storage volumes and associated costs. A recent report discusses hydrogen storage costs in Europe,^20^ but sources of these costs and the equations used by the study are missing. At the same time, learning experiences are not considered. This highlights the need for a comprehensive European-scale study of capacity assessment and cost analysis. Here, we calculate for the first time the technical hydrogen storage capacity of all existing gas storage sites in Europe, the associated costs, and predict future costs based on the learning experience. We consider site-specific hydrogen storage volumes and geological characteristics to calculate the capital costs required to upgrade the onshore UGS infrastructures. We also determine the levelized costs over the lifetime of the storage sites based on operational expenses. Additionally, we incorporate technology learning experience to project future levelized costs of hydrogen storage. Using this information, we identify UGS facilities with low-levelized costs of storage and a favorable capital cost to storage capacity ratio. This method enables us to characterize the performance of UHS sites in Europe and determine the most attractive sites for an early transition to hydrogen storage. Such a study is of importance to academia, industry, stakeholders, and policymakers for decision-making in the field of hydrogen energy.

## Methodology

First, we compile reservoir information of UGS sites in Europe and calculate the storage capacity of hydrogen in existing methane reserves using the Peng-Robinson equation of state. Next, we calculate the cost of transforming current UGS sites to hydrogen stores, taking into consideration the existing well infrastructure and storage site properties. Finally, recognizing that not all sites may be immediately suitable for hydrogen storage, we also project future costs utilizing Wright’s law. This predictive model leverages the concept of technological learning and experience to anticipate potential cost reductions as hydrogen storage technology advances and becomes more efficient over time.

As renewable hydrogen is currently a scarce resource, blending hydrogen with natural gas was suggested.^19^ When repurposing UGS for hydrogen storage, methane can be stored alongside hydrogen. The precise proportion of gases remains a matter of debate.^21^ Numerous studies propose that a hydrogen blend of approximately 10%–20% by volume (vol %) is acceptable for various end uses.^22^^,^^23^ For storage sites, it has been shown that up to 100% hydrogen by volume can be stored.^24^ Thus, we calculate results for 10%, 20%, and 100% hydrogen.

### Calculating storage capacity

The technical hydrogen storage capacity of UGS sites is calculated using site-specific reservoir volume and hydrogen density based on reservoir temperature and pressure. Data on European UGS sites used in the study were acquired from multiple sources. The three primary sources of data are the annual report of Oil and Natural Gas in the Federal Republic of Germany (LBEG),^4^ the International Gas Union (IGU),^25^ and Gas Infrastructure Europe (GIE).^3^ Although GIE has a comprehensive list of gas storage sites all over Europe, it only lists the working gas energy (WGE) for all the sites with no site-specific details about the depth, temperature, working gas volume, and storage pressure of the gas reservoirs. These parameters are essential for calculating the WGE of hydrogen-methane mixtures. Thus, we include this information from LBEG and IGU datasets. In cases of missing reservoir information in LBEG and IGU data, we referred to data published by the operating company to perform our calculations.

#### Density of gas at pressure-temperature conditions

Reservoir-specific hydrogen and methane densities were calculated using the Peng-Robinson equation^26^ of the state, which predicts the compressibility (Z) and density (ρ) of gases,^27^ given the acentric factor of gases (see the STAR methods section for details). We assume the surface temperature and pressure to be 15°C^28^ and 1 bar, respectively. Reservoir temperature and pressure conditions are used to calculate reservoir hydrogen and methane densities. If reservoir temperature is unavailable, we assume a 25°C/km geothermal gradient to calculate reservoir temperature from depth information.

#### Working gas energy

The energy content of the gas volume that can be obtained from a storage site during a cycle of injection and production is referred to as working gas energy (WGE). The corresponding volume is the working gas volume (WGV). We calculate the working gas energy (WGE) by using the reported maximum working gas volume (WGV) of the UGSs as a high-end estimate of WGE. This methodology does not involve as many assumptions as other approaches for WGV estimations,^29^^,^^30^ where reservoir thickness, radius, and areal extent are used for calculating WGV.

The WGE of the H_2_-CH_4_ mixture is a combination of the volumetric contribution of H_2_ and CH_4_ components. The WGE of the mixed gas can be written as^13^:

(Equation 1) $${WGE}_{mix}={lhv}_{H_{2}}\rho_{H_{2}}^{r}\left[V_{H_{2}}^{r}\left(\frac{\rho_{{CH}_{4}}^{S}}{\rho_{CH_{4}}^{r}}\right){WGV}_{{CH}_{4}}^{S}\right]+{lhv}_{{CH}_{4}}\rho_{CH_{4}}^{r}\left[V_{CH_{4}}^{r}\left(\frac{\rho_{{CH}_{4}}^{S}}{\rho_{CH_{4}}^{r}}\right){WGV}_{{CH}_{4}}^{S}\right]$$

where ${lhv}_{H_{2}}$ and ${lhv}_{{CH}_{4}}$ are the lower heating values of hydrogen and methane. The superscripts “*r*” and “*s*” indicate reservoir and surface conditions, respectively. For example, $\rho_{{CH}_{4}}^{S}$ and $\rho_{CH_{4}}^{r}$ are methane densities at reservoir and surface conditions, respectively. $V_{H_{2}}^{r}$ and $V_{CH_{4}}^{r}$ in Equation 1 are the volume fraction of hydrogen and methane at reservoir conditions, calculated as

(Equation 2) $$V_{H_{2}}^{r}=\frac{\left(\frac{\rho_{H_{2}}^{S}}{\rho_{H_{2}}^{r}}\right)V_{H_{2}}^{S}}{\left(\frac{\rho_{H_{2}}^{S}}{\rho_{H_{2}}^{r}}\right)V_{H_{2}}^{r}+\left(\frac{\rho_{CH_{4}}^{S}}{\rho_{CH_{4}}^{r}}\right)V_{CH_{4}}^{S}}$$

### Calculating H_2_ capital costs

Capital investments in both surface and subsurface infrastructure are necessary to store hydrogen in a UGS facility.^12^^,^^30^ Surface infrastructure includes equipment such as compressors, whereas subsurface infrastructure includes wells.^31^ A portion of the capital costs associated with subsurface hydrogen storage is attributed to working gas (H_2_) and cushion gas (H_2_ and CH_4_). Cushion gas refers to the volume of gas that remains in the storage reservoir during injection-production cycles.^32^ This volume of gas enables high reservoir pressure, which is necessary for the production of the working gas.^33^ Although site construction can be another major investment, we exclude this component from our capital cost analysis because we consider facilities that are already used for gas storage.

#### Surface infrastructure capital costs

The primary component contributing to surface capital costs is compressors and their associated infrastructure. The number of compressors required is determined by two key factors: the volume of stored hydrogen, measured as the working gas mass (WGM in kg), and the available time for injection. At present, typical compressors can compress approximately 2000 kg of hydrogen per hour to achieve the required reservoir pressures.^30^ For the purpose of our calculations, we make the following assumptions: (1) we assume that injection occurs for approximately four months of the year when excess renewable energy is available,^34^ i.e., approximately 2900 h. (2) We assume that the gas in porous media is used for seasonal storage,^9^ i.e., gas is cycled once a year,^7^ whereas, in salt caverns, gas is cycled monthly for four months a year.^35^ Thus, four times more gas needs to be compressed for salt cavern storage. To estimate the costs associated with compressors, we use a standard figure of $10.2 million per compressor.

(Equation 3) $$Compressor\;capital\;cost=\lceil \frac{Working\;Gas\;Mass\;\left[kg\right]}{Hours\;of\;operation\;\left[hr\right]\;x\;Compressor\;size\;\left[kg/hr\right]}\rceil x\;Compressor\;costs\;\left[\$\right]$$

The first term in Equation 3 is the ceiling absolute number of compressors.

#### Subsurface infrastructure capital costs

Assuming that one well can hold 3000 tons of hydrogen,^30^ new wells are required in some gas facilities, whereas in other facilities, the existing wells suffice. Facilities that require new wells incur more well costs, as new H_2_ wells are more expensive than retrofitting CH_4_ wells for H_2_ (Equations (Equation 4), (Equation 5), (Equation 6)). The cost of the well string increases with its length; therefore, deeper wells incur higher costs (Table 1). Note that this approach does not take site-specific reservoir injection properties into account.

(Equation 4) $$Number\;of\;required\;wells=\lceil \frac{Mass\;of\;hydrogen\;\left[kg\right]}{Capacity\;of\;each\;well\;\left[\text{kg}\right]\;}\rceil$$

**Table 1:** Various components of the capital cost of storage

Capital cost	Name of capital cost	Depleted gas field	Salt cavern	Saline aquifer
Gas cost	H_2_ cost ($/kg H_2_)	4	4	4
	Cushion gas %	50	20	80
	Hydrogen % in cushion gas	20	100	20
Well capital cost	New well cost ($/km/well)	1,280,000	1,280,000	1,280,000
	Retrofitting well cost ($/km/well)	290,000	290,000	290,000
Compressor capital cost	Total hours of operation (hours/year)	2900	2900	2900
	Compressor size (H_2_ kg/h)	2000	2000	2000
	Capitol cost per compressor ($)	10,200,000	10,200,000	10,200,000
	Compressor capacity (k ton H_2_)	11.2	11.2	11.2
	Compressor power (kWh/kg H_2_)	2.2	2.2	2.2
	Cost of electricity ($/kWh)	0.14	0.14	0.14
	Water requirement (L/kg H_2_)	50	50	50
	Water and cooling cost ($/100 L H_2_O)	0.02	0.02	0.02

We take the ceiling absolute value to be the number of required wells.

(Equation 5) Numberofnewwells=(Numberofrequiredwells−Numberofoldwells)

(Equation 6) $$Well\;capital\;cost\;\left[\$\right]=\left(Number\;of\;new\;wells\;x\;New\;well\;cost\left[\frac{\$}{m}\right]+Number\;of\;old\;wells\;x\;Old\;well\;cost\;\left[\frac{\$}{m}\right]\right)x\;depth\;\left[m\right]$$

#### Working and cushion gas capital costs

The cost of working gas, i.e., hydrogen, varies depending on how hydrogen is prepared.^36^ Green or renewable hydrogen, generated via water electrolysis powered by solar or wind energy sources, is most carbon neutral among other ways to generate hydrogen. Renewable hydrogen currently costs between $3 and $6 per kg H2.^37^^,^^38^ This resource plays a significant role in all eight of the European Commission’s outlined strategies for attaining net-zero emissions by the year 2050^1^^,^^39^; therefore, we consider the current cost of working gas to be an average of the reported green hydrogen cost, $4.^30^^,^^36^^,^^38^

Cushion gas needs are site-specific, with salt caverns having a higher working gas-to-cushion gas ratio than porous media stores^40^^,^^41^ (Table 1). Within the cushion gas volume, we consider 80% natural gas and 20% hydrogen for porous media and 100% hydrogen in salt caverns. The hydrogen fraction in cushion gas is essential for ease of separation of the working gas from the cushion gas. We argue that any natural gas used as cushion gas in all studied UGS would be still in place from previous operations, and thus, we do not assess costs associated with it.

Other capital costs include the costs for electricity as well as for water and cooling required to run the compressors the first-time hydrogen is stored in the reservoir. We assume an electricity price of $0.14 per kWh, an average of the last 10 years (based on^42^^,^^43^). We assume compressor power of 2.2 kWh per compressed kg of hydrogen, 50 L of water per compressed kg of hydrogen, associated with water and cooling costs of $0.02 per 100 L of water (Table 1).

(Equation 7) $$Electricity\;cost\;\left[\$\right]=WGM\left[kg\right]x\;Compressor\;Power\left[\frac{kWh}{kg}\right]x\;Cost\;of\;Electricity\;\left[\frac{\$}{kWh}\right]$$

### Water and cooling costs are calculated as

(Equation 8) $$Water\;and\;cooling\;cost\;\left[\$\right]=WGM\left[kg\right]\;x\;Water\;Requirement\left[\frac{L}{kg}\right]x\;Water\;and\;Cooling\;Cost\;\left[\frac{\$}{L}\right]$$

The total capital cost to store hydrogen in a reservoir is the summation of these three costs: working and cushion gas cost, well capital cost, and compressor cost. The numbers used for this calculation can be found in Table 1.

### Calculating H_2_ levelized costs

After making a capital investment in the hydrogen storage facility and storing hydrogen for the first time, levelized costs give an overview of the sustainability and maintenance of a storage site over the long term. Levelized cost accounts for the technical and economic costs occurring over the lifetime of the storage site. The technical aspect includes the storage capacity, whereas the economic aspect is dependent on capital investment, operational, and maintenance costs. Levelized costs for hydrogen storage (LCOS) are calculated as follows^30^:

(Equation 9) $$LCOS=\frac{Levelized\;TCC\;\left[\$\right]}{WGM\;\left[kg\right]}+Operational\;Cost\;\left[\frac{\$}{kg}\right]+Maintenance\;Cost\;\left[\frac{\$}{kg}\right]$$

Where levelized total capital cost (Levelized TCC) are

(Equation 10) LevelizedTCC={TCC.d(1+d)t(1+d)t−1}CF

where *d* is the discount rate, *TC*C is the total capital cost, *t* is the lifetime of the well, and *CF* is the capacity factor. Reduction in *d*, *TCC*, and *t* reduces the levelized total capital cost. We assume a capacity factor of 0.8, i.e., the plant is operated at 80% of the full-power operating potential in a year. The discount rate is assumed to be fixed for the lifetime of the well: 10% per year. We use a constant discount rate to protect from unexpected changes in the market. The yearly working gas capacity is higher in salt caverns than porous media storage due to higher cyclicity (monthly during the four months of excess energy); therefore, the denominator is higher in the first term of Equation 9 for salt caverns.

The second and third terms in Equation 9 are variable costs, which are used to calculate the average payment required to cover per-unit operational and maintenance costs. The operational and maintenance costs incurred from surface and subsurface infrastructure are adjusted from the literature^12^^,^^30^^,^^41^ and can be found in Table 2.

**Table 2:** Various components of levelized costs of storage

Levelized cost type	Name of levelized cost	Depleted gas field	Salt cavern	Saline aquifer
Levelized total capital cost	Discount rate (% per year)	10	10	10
	Well lifetime (years)	40	40	40
	Capacity factor	0.8	0.8	0.8
Compressor operation and maintenance cost	Electricity cost ($/kg H_2_)	0.31	0.31	0.31
	Water and cooling cost ($/kg H_2_)	0.01	0.01	0.01
Well operation and maintenance cost	H_2_ well cost ($/kg H_2_)	0.05	0.05	0.05
	H_2_ surface pipeline cost ($/kg H_2_)	0.0045	0.0045	0.0045

### Predicting future costs of H2 storage

The levelized cost of hydrogen in the future is based on the maturity of hydrogen storage technology.^44^^,^^45^ Because hydrogen storage technologies are in the earlier stages of development, the cost of hydrogen storage is relatively high. However, with the maturity of hydrogen technology, we expect a cost reduction in hydrogen storage technologies.^44^^,^^46^^,^^47^ The rate at which technologies mature is defined by the term “learning rate.” We assess the future cost of hydrogen storage of all UGS sites by assuming a learning rate (*LR*) of 15%.^45^

The learning curves for hydrogen storage are estimated based on Wright’s law as follows^46^:

(Equation 11) $$C_{f}=C_{in}{\left(\frac{X}{X_{in}}\right)}^{B}$$

(Equation 12) $$LR=1-2^{B}$$

where $C_{in}$ and $X_{in}$ are the initial capital expenditure and initial installation, respectively. $C_{f}$ is the capital expenditure at a given time in the future when the cumulative installation is X. We use Equations 11 and 12 to calculate the levelized cost of storage. Note that the projected costs of storage do not take inflation into account and that all costs are in 2023 US dollars.

## Results

### Hydrogen density

**Figure 1:**
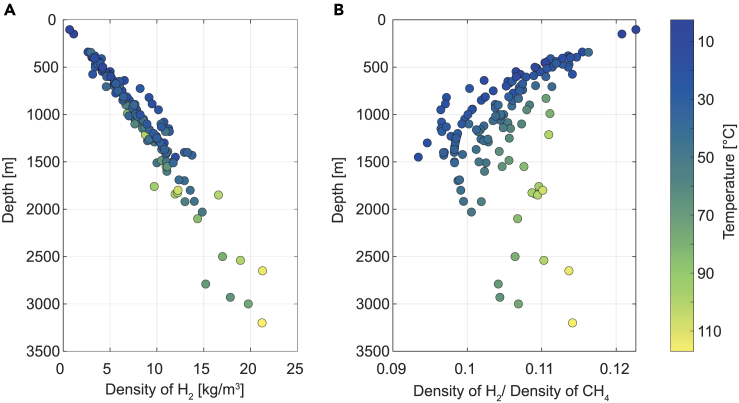
Density of hydrogen and methane with depth for all sites in Europe (A) Plot of reservoir depth with hydrogen density for the UHS sites shows that hydrogen density increases with increasing depth. The figure is color-coded by reservoir temperature. (B) Plot of reservoir depth with gas density ratio shows the lowest ratio at 1500 m. At the same temperature, the gas density ratio increases with increasing temperature.

Storage capacity depends on the volume of hydrogen stored, which in turn depends on the site-specific hydrogen density. Sites with high temperature and high pressure (deeper sites) have high hydrogen density (Figures 1A and S1). Hydrogen density evolves with depth by about 8 kg/m^3^ per kilometer (Figure 1A). Hydrogen is less dense than methane and the ratio of hydrogen density to methane density also depends on P-T conditions (Figure 1B). $\rho_{H_{2}}/\rho_{{CH}_{4}}$ decreases with depth till 1500 m. At depths higher than 1500 m, $\rho_{H_{2}}/\rho_{{CH}_{4}}$ increases.

At the same depth conditions, $\rho_{H_{2}}/\rho_{{CH}_{4}}$ increases with increasing temperature, resulting in higher storage capacities. Pressure has a more direct control on hydrogen density than temperature (see Figures S1A and S1C). When $\rho_{H_{2}}/\rho_{{CH}_{4}}$ is plotted with pressure, we observe an increase in the ratio up to 15 MPa pressure (Figure S1B). Above 15 MPa and 37°C, $\rho_{H_{2}}/\rho_{{CH}_{4}}$ increases with pressure and temperature (Figures S1B and S1D).

When hydrogen is stored in place of natural gas, the WGE is significantly lower due to the lower volumetric energy density of hydrogen.^48^ The ratio of hydrogen to methane density shows that given the same volume of the reservoir, less mass of H2 can be stored at 1500 m compared with depths above and below. Methane reservoirs at depths shallower than 1500 m can store more H2 by mass compared with UGS sites that are deeper.

A plot of pressure with depth for all UGS sites of Europe shows that most sites have hydrostatic pressure (Figure S2). Sites at high depths (>2 km) deviate from the hydrostatic gradient.

### Hydrogen storage capacity and working gas energy

The total WGE of hydrogen in all subsurface storage sites is about 25% of the total WGE of methane (Table S2). If all existing gas storages are replaced by hydrogen, the total amount of WGE that could be stored in countries of the European Union (EU) would be 260 terawatt hours (TWh). If non-EU countries are included, the total WGE would increase to 349 TWh (Table S1). The majority of existing storage sites (88%) have a low WGE (≤ 4 TWh WGE H_2_; Figure S3).

**Figure 2:**
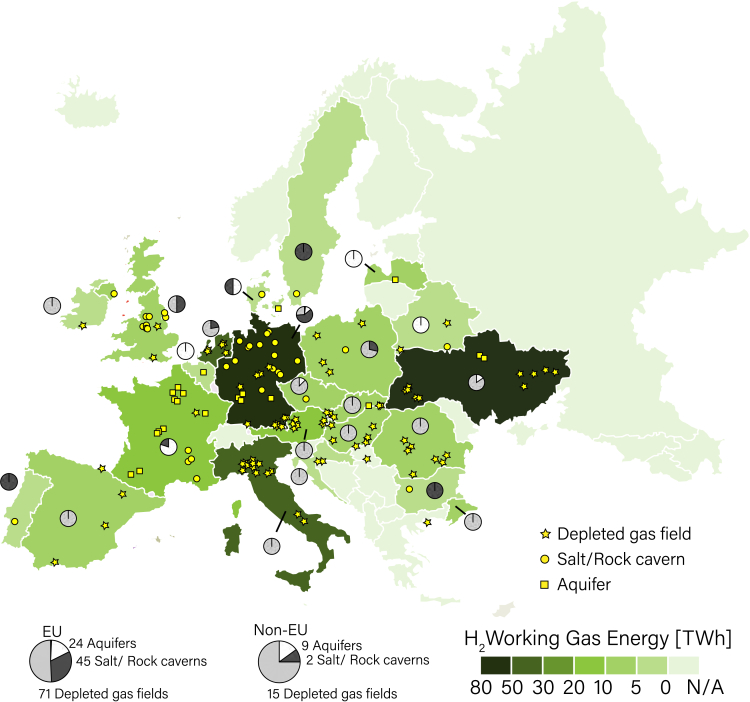
Hydrogen storage capacity of underground gas storage facilities in Europe per country (color scale) These facilities are categorized into (1) depleted gas fields, (2) salt caverns, and (3) aquifers. The pie circles for each country show the relative percentage of each field type (type-specific UGS numbers in Table S1).

Among the countries in the European Union, Germany has the largest number of UGS sites and a high hydrogen storage potential (Table S2), hosting approximately 29% of the WGE in the EU (71 TWh). Other EU countries with high hydrogen storage potential are the Netherlands and Italy, each comprising 20% (47 TWh) and 18% (37 TWh) of the total EU WGE, respectively. In the non-EU countries, Ukraine and the United Kingdom have the majority of the storage sites and high H_2_ WGE (Figure 2).

Depleted gas fields hold about 68% and 171 TWh of the total theoretical hydrogen storage capacity in UGS in the European Union (EU). Salt caverns can store up to 49 TWh (18%), and aquifers can only store up to 40 TWh of hydrogen (14%). Outside the EU, depleted gas fields have a storage capacity of up to 83 TWh (93% of total non-EU hydrogen storage potential).

### Costs of H_2_ storage in Europe

**Figure 3:**
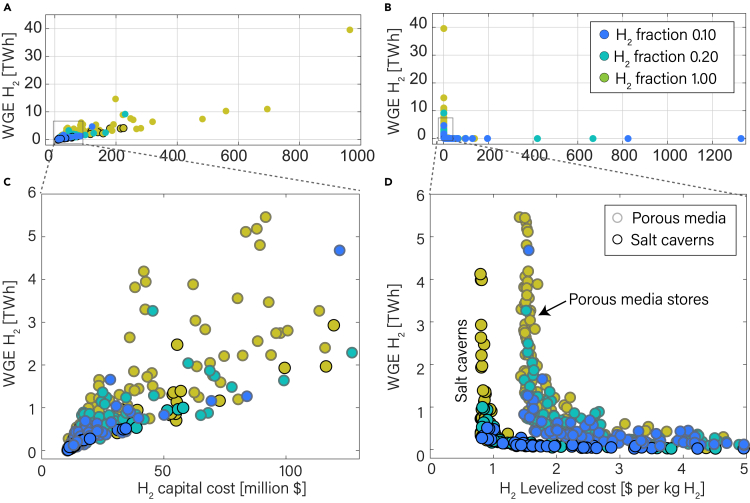
Capital and levelized costs vary with storage capacity (A) Capital cost required to transform all studied underground gas storage (UGS) sites to hydrogen storage sites shows a positive correlation with the working gas energy of hydrogen (WGE H_2_). Higher capital costs are required with increasing hydrogen fraction, i.e., 0.1 (10%), 0.2 (20%), and 1 (100%) H_2_ fraction. (B) Levelized cost of storage (LCOS) as a function of WGE H_2_ has an L-shape, i.e., LCOS is high at low WGE H_2_. (C) Zoomed view of (A), only showing the sites with WGE H2 below 6 TWh. Onshore salt caverns (bold edges), in which more compressors are required for annual cycling, have higher capital costs at the same WGE compared with porous media stores. (D) A zoomed view of 3b shows that salt caverns have lower LCOS than porous media stores for the same WGE H2. If WGE H_2_ is more than 0.5 TWh, then LCOS approaches a lower limit of ∼$0.8 per kg H_2_ for salt caverns (bold edges) and about ∼$1.5 per kg H_2_ for depleted gas fields and aquifers.

Based on our economic analysis, the capital costs of underground hydrogen storage range from $10 million for small storage sites to $1 billion for large storage facilities (Figure 3A). Eighty-three percent of the stores have capital costs of less than $100 million (Figure S4). Levelized costs (LCOS) of H_2_ storage, which include the levelized capital costs as well as operational and maintenance costs over the lifetime of a storage site, are high (>$2 per kg H_2_) for storage facilities with low WGE (Figure 3B). LCOS decreases sharply with an increase in stored energy, reaching a plateau for sites above 0.5 TWh WGE (Figure 3D). The main reasons are the high capital costs for compressors and well-retrofitting, as well as associated maintenance and operational operating costs, which are needed for all sites.

There is a distinct difference in costs for salt caverns and porous media storage sites. Gas storage caverns are developed for hydrogen demand support.^41^^,^^49^ Typically, a production-injection cycle for a porous media store occurs over the course of one year, whereas in salt caverns hydrogen can be cycled even more frequently. Salt caverns can achieve a recovery efficiency of 100%.^50^ This results in a higher need for compressors for salt cavern sites, resulting in higher capital costs (Figure 3C). The higher cycling rate results in a higher annual WGE, decreasing the levelized costs of salt caverns. The LCOS for most salt caverns is about $0.8 per kg H_2_, which is about half the LCOS of hydrogen storage in porous media, i.e., $1.5 per kg H_2_ (Figure 3D).

### H_2_ storage cost reduction in three experience cycles

Wright’s law uses current storage capacity (or installments) and current expenditure to calculate the cost of future installations. We identify current pilot sites. Current projects like H_2_Cast in Germany, Hypster^51^ in France, and Hystock^52^ in the Netherlands are working to store H_2_ in operational salt caverns: Etzel, Etrez, and Zuidwending, respectively (dark circles in Figure 6B). The experience of these salt cavern stores in the last decade has improved our technology knowledge. Thus, we assume that salt caverns will be used first.

Development of depleted gas fields by Underground Sun Storage^53^ in Austria and Teréga’s aquifers in France are expected to store hydrogen by 2030. With this experience, porous media storage sites can be used alongside salt caverns shortly (after 2030).

**Figure 4:**
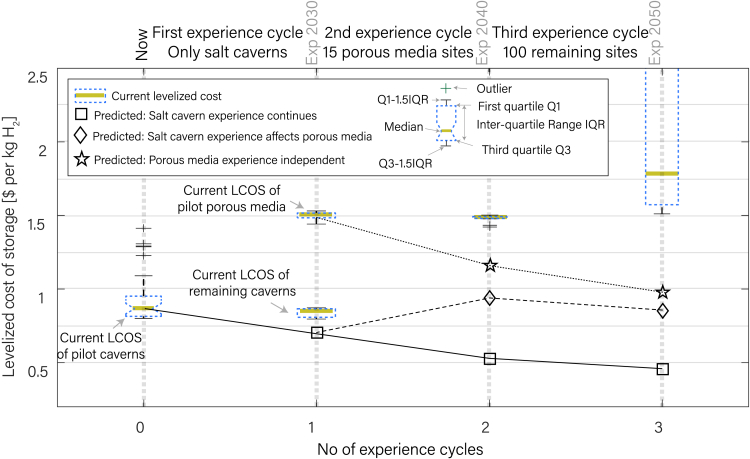
Temporal evolution of levelized cost of hydrogen storage with experience cycles This plot compares the current LCOS, shown by boxplots, with predicted future LCOS for three different scenarios, shown by squares, diamonds, and stars. In the first scenario (squares), experience gained from salt cavern reduces LCOS of porous media storage. In the second scenario (diamonds), experience gained from salt cavern partially affects the LCOS of porous media, whereas, in the third case (stars), experience of porous media is independent of salt caverns. Reduction in LCOS is most pronounced when experience from salt cavern can be entirely carried over to porous media storage. For Cases 1, 2 and 3, the LCOS reduces to $0.4, $0.8, and $1 per kg H_2_ at the end of the three experience cycles.

We predict future LCOS for three scenarios. Experience gained from hydrogen storage in salt caverns can (1) fully (2) partially, or (3) not at all be transferred to porous media storage (Figure 4). In Case 1 (squares), the experience acquired from the current pilot projects of salt caverns is transferrable to salt caverns and porous media stores in the upcoming experience cycles. Case 2 (diamonds) and Case 3 (stars) start from the first experience cycle because currently there is no experience storing hydrogen in porous media in any of the European sites. For Case 2, the experience of the salt cavern is transferable to porous media, i.e., the LCOS from the salt cavern after the ongoing experience cycle is the predicted LCOS of the mixed case. LCOS for Case 3 is independent of the salt cavern experience.

According to our calculations, the currently active salt cavern projects will have a total WGE of 13 TWh with an average LCOS of $0.8 per kg H_2_. The first experience cycle of repurposing all salt caverns decreases the LCOS to $0.7 per kg H_2_. The second experience cycle can utilize techno-economically suitable porous media sites. If experience gained from the first salt cavern storage cycle is completely transferable to porous media storage (Case 1), LCOS decreases from $0.5 to $0.4 per kg H_2_ during the second experience cycle. Instead, if the experience is partially transferable (Case 2), the LCOS after the second cycle is $0.9 per kg H_2_. If no experience is transferable from salt cavern storage to porous media storage (Case 3), LCOS decreases from $1.5 per kg H_2_ to $1.2 per kg H_2_. If the rest of the storage sites are used by the third experience cycle, LCOS for Cases 1, 2, and 3 will be $0.4, $0.8, and $1.0 per kg H_2_, respectively. Such systematic implementation of UGS facilities for hydrogen storage reduces LCOS. The time required to achieve the experience rate is not predictable. The need for storage of pure H_2_ is expected to arise in the next 10 to 20 years, but the areas of application and projections are still uncertain. This ambiguity hinders long-term investment decisions and causes further delays in developing business cases. We anticipate that these three cycles of experience will persist until 2030, 2040, and 2050, respectively, provided that technical proficiency continues to evolve as required.

## Discussion

### Effect of hydrogen-methane blends

With increasing hydrogen fraction in the hydrogen-methane blend, we observe an increase in the WGE H2 and an increase in the capital cost of storage. This can be observed in Figures 3A and 3C, where green dots plot more to the right. The trend of LCOS with hydrogen-methane blend in Figure 3D is such that LCOS is higher at low hydrogen fractions and lower at high hydrogen fractions. This can be seen from the blue dots, which have higher LCOS.

**Figure 5:**
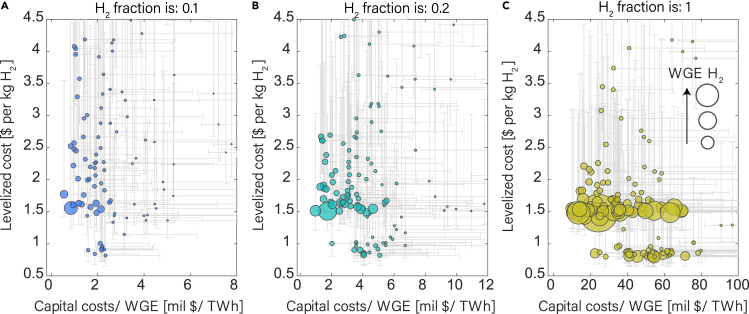
Ratio of capital cost to total WGE and LCOS are used for site selection The figure shows three hydrogen fractions (A) 0.1, (B) 0.2, (C) 1. (A–C) Cost uncertainties are shown by the error bars (gray lines). Capital and levelized cost uncertainties are represented by standard deviations of 10% and 20%, respectively. The size of the circle quantifies the WGE of H_2_. The x and y axes limits of these figures are limited to clearly distinguish between the porous media and salt cavern sites.

Due to the low energy density and higher working gas volume of hydrogen compared with methane, it is easier to compare the cost per unit of total energy (WGE H_2_ + WGE CH_4_). We therefore show the capital cost per unit of total WGE for the three different H_2_-CH_4_ blends in Figure 5. We observe that high methane content in the working gas increases the WGE, and the capital costs associated with the retrofitting decrease, as less hydrogen gas is needed (Figures 5A–5C). However, the LCOS increases, as retrofitting capital and levelized costs still occur.

The concept of mixing hydrogen with natural gas was suggested as a temporary measure to establish a complete production, storage, transmission, and utilization chain for hydrogen.^54^ In terms of subsurface storage, hydrogen-methane blends can be stored in the same reservoir, resulting in a higher WGE storage compared with pure hydrogen storage (as shown in Figure 4). Additionally, by introducing hydrogen into combustion processes, CO2 emissions can be lowered while preserving energy value and diminishing the carbon footprint.^21^^,^^55^ Furthermore, integrating hydrogen into heat and electricity generation can provide similar benefits to those of renewable energy sources, which can further bolster sustainability efforts. Due to the limited availability and high costs of renewable-based hydrogen, as well as the lower WGE and high capital costs of hydrogen storage sites, natural gas combined with carbon capture and storage (CCS) can also be an option for seasonal, energy-security-focused storage. However, there are obstacles associated with extracting hydrogen from these blends, such as hydrogen embrittlement,^41^^,^^56^ which necessitates the implementation of safety measures. Pressure drops^57^ and energy delivery issues^58^ can also arise as a result of hydrogen’s lower energy density, making network planning a critical component. Adapting combustion systems to accommodate varying hydrogen percentages presents a challenge, and utilizing hydrogen for residential heating is not recommended.^59^ Safe hydrogen concentration levels are determined by the blend’s hydrogen content, with lower blends posing fewer concerns than higher blends. For higher blends, safety protocols and equipment modifications are required.^60^ Moreover, blending hydrogen with methane for storage would require separation and cleaning of the gases after production, which costs above $4 per kg H_2_.^48^ Thus, it increases the overall cost of hydrogen, if pure hydrogen is needed for industrial applications.

### Site selection based on techno-economic aspects

There are many aspects that can be considered for site selection for hydrogen storage.^61^^,^^62^^,^^63^^,^^64^ Assuming that the main factors for site selection are the costs and energy storage capacity, we can identify UGS sites that are best suited for conversion to hydrogen storage sites. Although salt caverns have lower storage capacity than depleted gas reservoirs and saline aquifers^65^ (Figures 3A and 3C), we predict that salt caverns will be the most suitable type of storage for their low-levelized cost (Figures 3D and 5A–5C). The impermeable salt layer provides a natural barrier to prevent leaks; therefore, annual leakage is lower in salt caverns.^66^ Evaporite materials in salt, including anhydrite, gypsum, and rock salts, provide exceptional sealing capabilities and redistribute stress through viscous-plastic deformation.^67^ Moreover, salt caverns' high salinity environment makes them resistant to microbial activity.^68^^,^^69^ Artificial caverns have flexibility of shape and size.^15^ Compared with depleted gas fields and aquifers, which may experience unpredictable changes in pressure and temperature that could affect hydrogen stability, salt caverns offer greater stability.^7^^,^^9^^,^^67^

**Figure 6:**
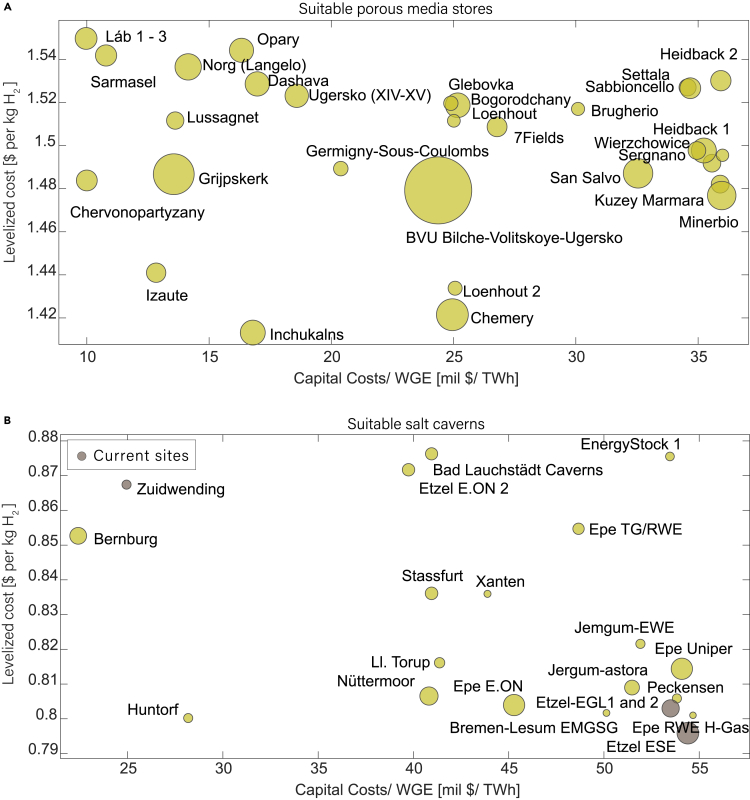
Best sites for hydrogen storage based on techno-economics (A) Zoomed version of Figure 5C for porous media stores and corresponding facility/location name. Sites with low capital cost to WGE and low LCOS are best suited for storage. (B) Zoomed version of Figure 5C for salt caverns with low capital cost to WGE and low LCOS. The levelized cost is almost similar, so the ones with lower capital cost/working gas energy ratio are best suited for hydrogen storage. Current sites with pilot project studies are in dark green.

Several hydrogen storage projects are currently being implemented in salt caverns in Europe. Therefore, there is more practical experience for hydrogen storage.^65^ Among the salt cavern UGS sites, those with low levelized costs and a low capital cost to working gas energy (WGE) ratio are the most feasible for hydrogen storage: Huntorf, Bernburg, Catherine, Stassfurt, Nuttermoor, Epe, and Etzel stores located in Germany (Figure 6B). Porous media storage sites that are techno-economically most favorable are Grijpskerk, Norg, Netherlands; Inchukalns, Latvia; Chervonopartyzany, Dashava, Opary, Bilche-Volitskoye-Ugersko, Ukraine; and Izaute, Lussagnet, Germigny-sous-Coulombs, France (Figure 6A).

Given these recommendations, the design and operation of a hydrogen storage facility will be driven by specific investment targets. Other factors may include the expected demand for hydrogen, the regulatory environment, and also the availability of funding.^45^

### Uncertainties in forecasting levelized costs

We show that repurposing the existing UGS sites while gaining experience can reduce the future LCOS by up to 50% ($0.8 to $0.4 per kg H2) within three experience cycles, suggesting a significant effect on the economy of scale. There is a general recognition that long-term cost predictions for energy technologies are uncertain and strongly influenced by assumptions of how quickly the technology will improve^46^; therefore, LCOS at the first salt storage experience cycle is more certain than later ones. The growing industry and governmental funding in porous media storage can also increase the learning rate beyond 15% by 2030, which will also reduce the overall cost projection. Likely, some of the experience gains predicted to lower future levelized costs of storage will be nullified by inflation.

The motivation for energy storage, whether it be for short-term supply-demand regulations or seasonal storage, is a crucial factor to consider when designing a storage site. Although high cyclicity is economically advantageous, as evidenced by the lower levelized cost of storage (LOCS) for salt caverns, the political need for seasonal energy security may serve as a primary driver for the development of storage sites. The LCOS will also be determined by the energy demand. Decreasing the energy demand by 10% can reduce the LCOS in salt caverns by 9%.^45^ However, due to the growing population, lifestyle changes, and the need for space cooling, the energy demand is expected to even increase by 15% by 2050. An increase in energy demand would increase the LCOS beyond our predicted LCOS.

### Technological perspective

In this study, the repurposing of pre-existing UGS sites for hydrogen storage is assessed, revealing a total current potential for 349 TWh in the existing UGS sites of Europe. This falls at the lower end of predicted hydrogen storage needs in 2050, which range between 150 and 1500 TWh and are highly dependent on the assumptions made in the scenarios (Figure S5). Currently, seasonal energy storage within the EU is about 1335 TWh of natural gas (mandatory 90% filling level before winter periods). This indicates that only retrofitting existing UGS for hydrogen storage will not be sufficient for seasonal energy security.

From a technological perspective, repurposing salt caverns for hydrogen storage is feasible due to the existing knowledge and experience. However, utilizing porous media stores for hydrogen storage presents several challenges, as experience in this area is still lacking. Bio-geochemical reactions within the storage reservoir can occur, leading to hydrogen consumption and gas contamination, which may impede storage operations. The diffusion of hydrogen into the caprock above the storage reservoir may result in losses of hydrogen from the storage cycle.^70^ If methane is present in the caprock—which is often the case when repurposing UGS—this diffusion may be significantly increased.

The migration of hydrogen out of the storage reservoir would negatively affect storage security (see Section S6). The ability of the caprock to trap hydrogen within an underlying reservoir depends on the multi-phase flow properties of the hydrogen-brine-rock system. At depths below about 1100 m, there are indications that the potential for structural trapping decreases^71^ (Figure S6). This means that the same reservoir is not capable of holding similar amounts of hydrogen as it currently holds methane. A preliminary assessment of two porous media stores in the Czech Republic shows that some porous media UGS sites are unlikely to be able to store the same amount of hydrogen as working gas based on wettability conditions (Figure S7). In current hydrogen storage scenarios, porous media stores often play a major role, but the security of these sites has to be further validated.

### Economic perspective

The capital and levelized costs of hydrogen storage presented earlier are in the same order of magnitude as previously reported.^30^^,^^72^^,^^73^ LCOS for fast cycling salt caverns is thought to be in the range of $0.14–2.2 per kg H_2_^72^^,^^73^ whereas LOCS for seasonal storage in depleted gas fields range from $1.2–2.5 per kg H_2_.^20^^,^^30^ The capital cost for the development of new storage sites has been estimated to range from $100s–1000 million for the Intermountain West region of the USA^30^ and from $600–1600 million for the EU.^20^

The cost of working gas depends on the process used to produce hydrogen and the allowances for CO2 emissions.^74^ Green hydrogen has no CO2 emission allowance and is the preferred choice for storage. However, other forms of produced hydrogen can also be stored underground to meet higher demand. "Blue" hydrogen, the next environment-friendly option of hydrogen energy, is believed to have the potential to rapidly reduce CO2 emissions and expedite the transition, especially by 2030.^75^ This transition is important as the capacity to supply "green" hydrogen from dedicated renewable energy sources is projected to be insufficient to meet the growing local and regional demand by that time. The projected production expenses for "blue" hydrogen are estimated to range from $1.4–2.0 per kg^76^ (assuming a euro to dollar conversation rate to be ∼1), assuming there are moderate natural gas and CO2 prices. However, it is anticipated that these costs could potentially rise to $1.6 to 2.3 per kg during the 2030s and 2040s, contingent on the rise of CO2 emission allowance prices.^75^^,^^76^^,^^77^ It is estimated that "blue" hydrogen could attain competitiveness in Europe if the cost of CO2 emission reaches the bracket of $0.050–0.06 per kg H_2_.^38^ By the year 2050, nearly 4,000 TWh of green hydrogen could be manufactured at prices below $2.0 per kg, with approximately 2,500 TWh available at rates under $1.5 per kg and approximately 600 TWh at $1.0 per kg.^76^ Keeping all technical aspects and other costs the same, such as reduction of working gas production cost from $4 to $2 or $1 will reduce capital cost by 2/3^rd^ or 1/2^th^, respectively.

### Future outlook

The process of repurposing UGS sites for hydrogen use, or constructing new hydrogen storage facilities, is time-consuming. Retrofitting an existing UGS site may require 1–3 years, whereas building a new salt cavern storage site can take up to 5–7 years, and developing a porous media storage site may take up to 10 years.^8^ Hence, if subsurface hydrogen storage is a significant part of the energy transition, site selection and repurposing or development must occur at least 10 years before the anticipated demand.

### Limitations of the study

Our site selection process^78^^,^^79^ focuses exclusively on storage capacity and cost considerations. To ensure consistency, we have employed parameters that are universally available across all sites, including factors such as pressure, temperature, working gas volume, and the number of existing wells for our calculations. Notably, we have not factored in other technical variables that may impact site selection, such as solid-fluid interactions encompassing adsorption-desorption, relative permeability, mobility, and their ratios. We have also not accounted for external factors influencing site selection, such as transportation grids, road and railway infrastructure, hydrogen demand, and societal acceptance of the technology. To be ready for substantial hydrogen production, each existing site must be investigated to store pure and blended hydrogen. Moreover, we anticipate that not all salt caverns, depleted gas fields, and aquifers will undergo conversion for hydrogen storage. A portion of the storage capacity is expected to be reserved for storing methane and biomethane. Some of these storage facilities may undergo conversion for the storage of CO₂, specifically for Carbon Capture and Storage (CCS) applications. It is worth noting that obtaining data on these aspects and quantitatively incorporating them into a continental-scale study poses significant challenges. These areas represent potential avenues for future research, where the creation of comprehensive databases can enhance the precision of site selection processes.

## STAR★Methods

### Key resources table

**Table:** 

REAGENT or RESOURCE	SOURCE	IDENTIFIER
**Software and algorithms**		

MATLAB 2022a	Mathworks	https://www.mathworks.com/products/matlab.html
Python v 3.11	Visual Studio Code	https://code.visualstudio.com/

### Resource availability

#### Lead contact

Further information and requests for resources should be directed to and will be fulfilled by the lead contact, Mayukh Talukdar (mayukh.talukdar@kit.edu).

#### Materials availability

This study did not generate new unique reagents.

#### Data and code availability

• This paper analyses existing, publicly available data. ALL data reported in this paper is shared in the supplementary material. We attach a supplementary Excel file 'site-specific technical and economic calculations'.
• This paper does not report the original code. The code for the analysis was written in MATLAB and is available from the lead contact upon request.
• Any additional information required to reanalyze the data reported in this paper is available from the lead contact upon request.

### Method details

Calculating the compressibility and density of gas at a given pressure, temperature, and volume

(Equation 13) $$P=\frac{RT}{V_{m}-b}-\frac{a\alpha }{V_{m}^{2}+2bV_{m}-b^{2}}$$

Where P and T are pressure and temperature, respectively. V_m_ is the molar volume, whereas R is the universal gas constant (8.3144 J/mol K). a, b, and α are constants and are defined as:

(Equation 14) $$a=\frac{0.45724\;R^{2}T_{c}^{2}}{P_{c}}$$

(Equation 15) $$b=\frac{0.07780\;R\;T_{c}}{P_{c}}$$

(Equation 16) $$\alpha ={\left(1+\left(0.37464+1.54226\;\omega -0.26992\;\omega^{2}\right)\left(1-T_{r}^{0.5}\right)\right)}^{2}$$

(Equation 17) $$T_{r}=\frac{T}{T_{c}}$$

The acentric factor (ω) of methane and hydrogen used for these calculations are 0.011 and −0.216, respectively.^80^ Methane’s critical temperature (Tc) is 191.15 K, whereas the critical pressure (P_c_) is 46.1 bars.^9^ The critical temperature of hydrogen is 33.18 K, whereas the critical pressure is 13 bars. In polynomial form, we can write:

(Equation 18) $$A=\frac{a\alpha P}{R^{2}T^{2}}$$

(Equation 19) $$B=\frac{bP}{RT}$$

(Equation 20) $$Z^{3}-\left(1-B\right)Z^{2}+\left(A-2B-3B^{2}\right)Z-\left(AB-B^{2}-B^{3}\right)=0$$

(Equation 21) $$\frac{1}{V_{m}}=\frac{P}{RTZ}$$

Multiplying the molecular weight of gas with Equation 9 gives the gas density in given P-T conditions.

We calculate the density of methane and hydrogen at the P-T conditions of the UGS sites (site specific details in the attached supplementary excel sheet).

## Supplemental information

Document S1. Figures S1–S7 and Tables S1 and S2

Site-specific technical and economic calculations The file contains information about each site in Europe, input parameters used for the calculations, and the components of capital and levelized costs. The details about the calculation can be found in the Methodology section of the paper. The corresponding results for each site are shown in more details in Results and Discussion sections.
