# Supplementary material for: Techno-economic analysis of underground hydrogen storage in Europe
Source: iScience. 2023 Dec 20;27(1):108771. doi: 10.1016/j.isci.2023.108771 (PMC10821165; doi:10.1016/j.isci.2023.108771)
Supplement: Document S1. Figures S1–S7 and Tables S1 and S2 [file mmc1.pdf]

iScience, Volume *27*

Supplemental information

Techno-economic analysis of underground hydrogen storage in Europe

Mayukh Talukdar, Philipp Blum, Niklas Heinemann, and Johannes Miocic

S1. Underground Gas Sites in Europe

Table S1. Underground storage sites (UGS) in Europe and their storage types.

Country	Depleted gas field	Salt cavern	Aquifer	Total
Austria	9	0	0	9
Belgium	0	0	2	2
Bulgaria	0	1	0	1
Croatia	1	0	0	1
Czech Republic	7	0	1	8
Denmark	0	1	1	2
France	0	3	12	15
Germany	15	34	7	57
Hungary	5	0	0	5
Ireland	1	0	0	1
Italy	11	0	0	11
Latvia	0	0	1	1
Netherlands	7	2	0	9
Poland	5	2	0	7
Portugal	0	1	0	1
Romania	7	0	0	7
Slovakia	2	0	0	2
Spain	2	0	0	2
Sweden	0	1	0	1
**Total EU**	**72**	**45**	**24**	**142**
Belarus	0	0	1	1
Turkey	2	0	0	2
Ukraine	11	0	2	13
United Kingdom	2	2	0	4
**Total non-EU**	**15**	**2**	**3**	**20**
**Total EU and non-EU**	**87**	**47**	**27**	**162**

These country-wise underground storage types are shown as pie charts in Figure 2. The storage sites are compiled from multiple sources and can be found in the supplementary Excel sheet.

S2. Gas density in the UHS sites and their relationship to Pressure-Temperature

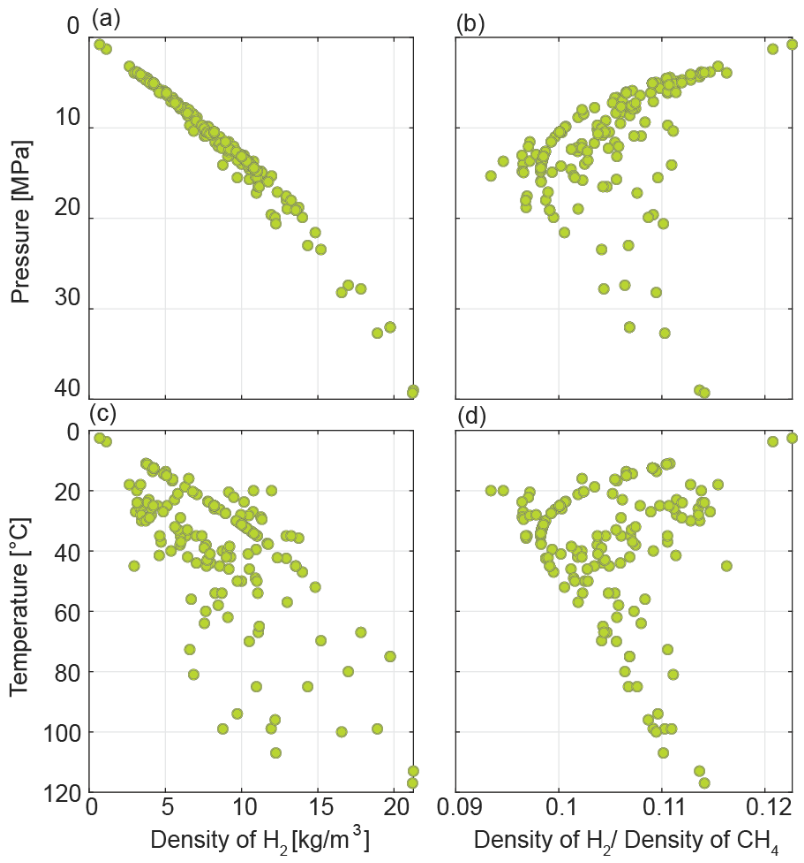

Figure S1. Density of hydrogen and methane calculated from the Peng-Robinson equation. (a) Plot of reservoir pressure with hydrogen gas density for the UHS sites showing how hydrogen density increases with increasing pressure. A similar plot with depth instead of pressure is Figure 1. (b) A plot of reservoir pressure with gas density ratio shows the lowest ratio at 15 MPa pressure. (c) Plot of reservoir temperature with hydrogen density. (d) Plot of reservoir temperature with gas density ratio.

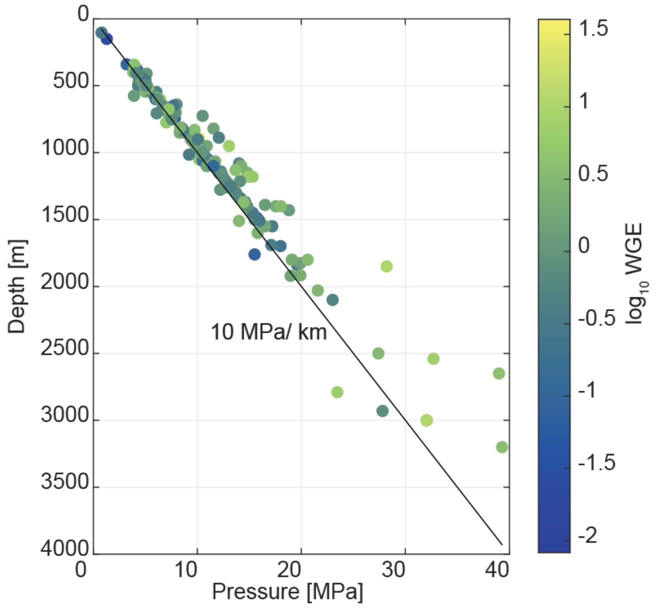

Figure S2. Pressure-Depth plot of all UGS sites of Europe. Most of the sites fall along the hydrostatic pressure gradient of 10 MPa/km. There are a few sites at higher depths, where overpressure can be observed. These sites have high WGE.

S3. Storage Potential in Europe

Table S2. Total and hydrogen working gas energy (WGE) in European underground storage sites for each country.

	CH_4=100%	CH_4: H_2=90: 10		CH_4: H_2=80: 20		H_2=100%
Country	***WGE Total***	***WGE Total***	***WGE H_2***	***WGE Total***	***WGE H_2***	***WGE H_2***
Austria	81.9	74.5	2.4	67.5	4.7	20.2
Belgium	13.0	11.8	0.4	10.7	0.7	3.2
Bulgaria	9.5	8.6	0.3	7.8	0.5	2.3
Croatia	5.3	4.9	0.2	4.4	0.3	1.4
Czech Republic	32.9	29.9	1.0	27.1	1.9	8.2
Denmark	7.8	7.1	0.2	6.4	0.4	1.9
France	115.6	105.6	3.4	96.0	6.7	29.6
Germany	284.8	259.4	8.4	235.0	16.4	70.7
Hungary	31.7	29.1	0.9	26.5	1.9	8.4
Italy	159.0	143.8	4.6	129.5	9.0	37.3
Latvia	21.8	19.9	0.6	18.1	1.3	5.6
Netherlands	179.8	164.8	5.3	150.2	10.5	47.0
Poland	18.6	16.9	0.5	15.4	1.1	4.6
Portugal	1.4	1.3	0.0	1.2	0.1	0.4
Romania	37.6	34.3	1.1	31.2	2.2	9.6
Slovakia	21.2	19.5	0.6	17.8	1.2	5.7
Spain	14.3	13.1	0.4	11.9	0.8	3.7
Sweden	0.1	0.1	0.0	0.1	0.0	0.0

**Total EU**	1037.1	945.5	30.6	857.5	59.9	260.0
Ireland	0.9	0.9	0.0	0.8	0.1	0.3
Belarus	3.8	3.5	0.1	3.2	0.2	1.0
Turkey	15.2	13.7	0.4	12.3	0.9	3.5
Ukraine	302.5	276.0	8.9	250.6	17.5	76.3
United Kingdom	31.2	28.5	0.9	25.8	1.8	7.8
**Total non-EU**	352.7	321.7	10.4	291.9	20.4	88.6
**Total EU and non-EU**	**1389.8**	**1267.2**	**41.0**	**1149.3**	**80.3**	**348.6**

The Working Gas Energy per country is color-coded in Figure 2 of the main document.

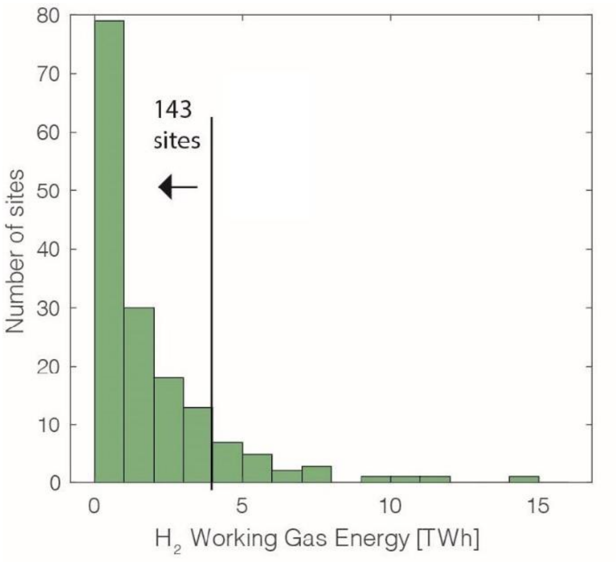

Figure S3. Histogram of working gas energy of 100% hydrogen. 143 out of 162 sites (88% of the sites) have WGE less than 4 TWh.

S4. Histogram of capital cost of hydrogen storage

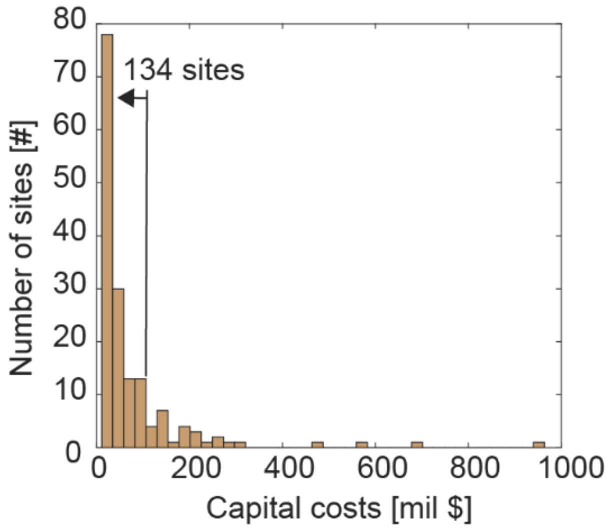

Figure S4. Histogram of the capital cost of hydrogen storage with 100% H_2. 134 sites (83%) of 162 sites require a capital cost of 100 million $. This number is discussed in the Results section on 'Cost of hydrogen storage in Europe'.

S5. Projected H2 demand, storage needed, and available

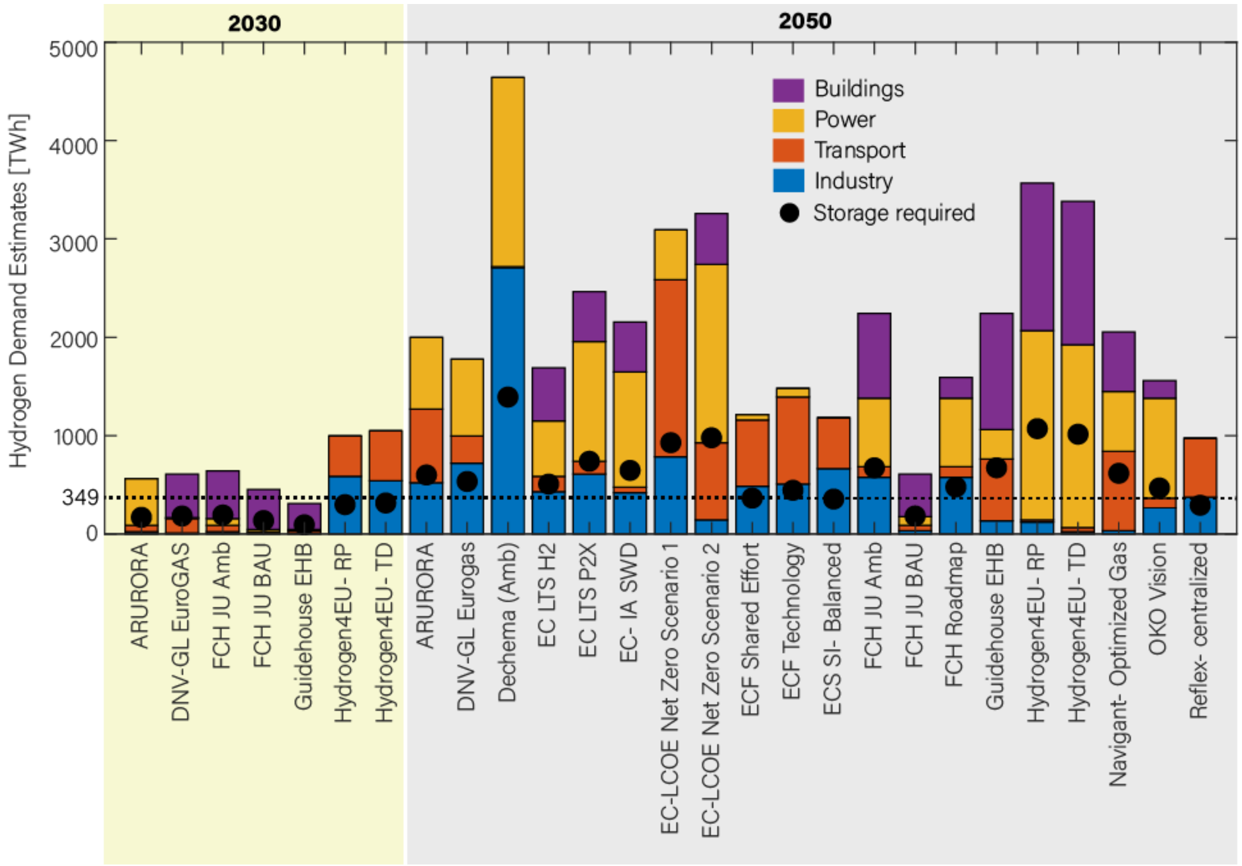

Figure S5. Current hydrogen storage potential compared with several demand estimates in 2030 and 2050 (AURORA[1], DNV[2], FCH[3], Guidehouse[4], Hydrogen4EU[5], Dechema[6], EC LTS[7], EC LCOE[8,9], ECF Shared Effort[10], EC SI[11], Navigant[12], Oko[13], Reflex[14]). Underground storage needs are 30% of the methane demand[15]: assuming the

same for hydrogen, we show the underground hydrogen need in black dots. The total storage available in existing underground hydrogen storage is shown by a dotted black line for comparison. This aspect is covered in the 'Technological perspective' subsection of the Discussion section.

S7. Structural trapping of hydrogen

Hydrogen is highly permeable and can leak through the storage facility's rock formations, which can lead to safety hazards and economic losses. Despite having low permeability, caprock still permits gases to percolate through it. However, the capillary forces within the caprock are strong enough to prevent the majority of buoyant gases from escaping into the atmosphere. The balance between capillary and buoyancy forces to determine the height of H_2 gas that can be effectively stored through structural trapping can be written as:

$$h = \frac{2\gamma\ cos\theta}{rg\Delta\rho}$$

The column height h depends on the H_2-brine interfacial tension γ, H_2-brine-rock contact angle θ, H_2-brine density difference $\Delta\rho$, gravitational constant g and pore radius of the caprock r[16].

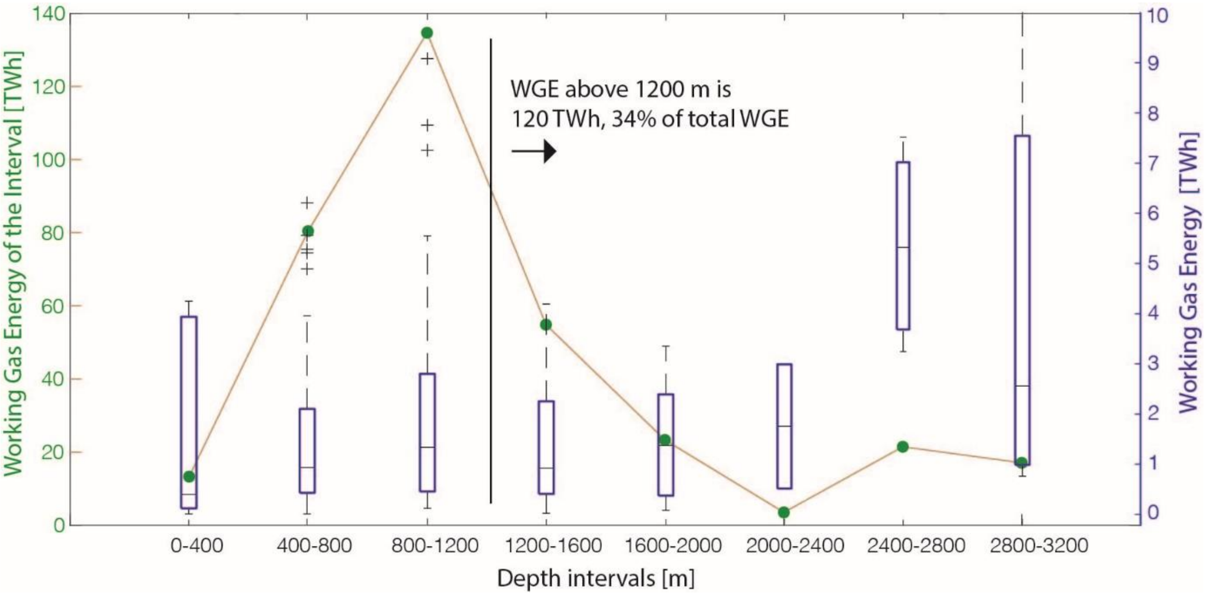

Figure S6. Total working gas energy plotted for sites of specific depth intervals. The working gas volume of deeper sites is lower than the calculated working gas because of wettability reasons.

We constrain the column of hydrogen that can be stored by using this method for two different gas fields in the Czech Republic: Uhrice and Zarosice gas fields[17]. The column height of CH_4 in Uhrice is almost the same as the permissible column height of H_2 at that depth; however, the column height of CH_4 at Zarosice is much larger than the column height of H_2. This suggests that the amount of hydrogen that can be stored in the existing gas storage sites is lower than the calculated WGV due to wettability conditions. Since the column of gas is smaller at greater depths, the hydrogen storage potential for deeper gas storage sites can be considerably lower than that estimated by WGV.

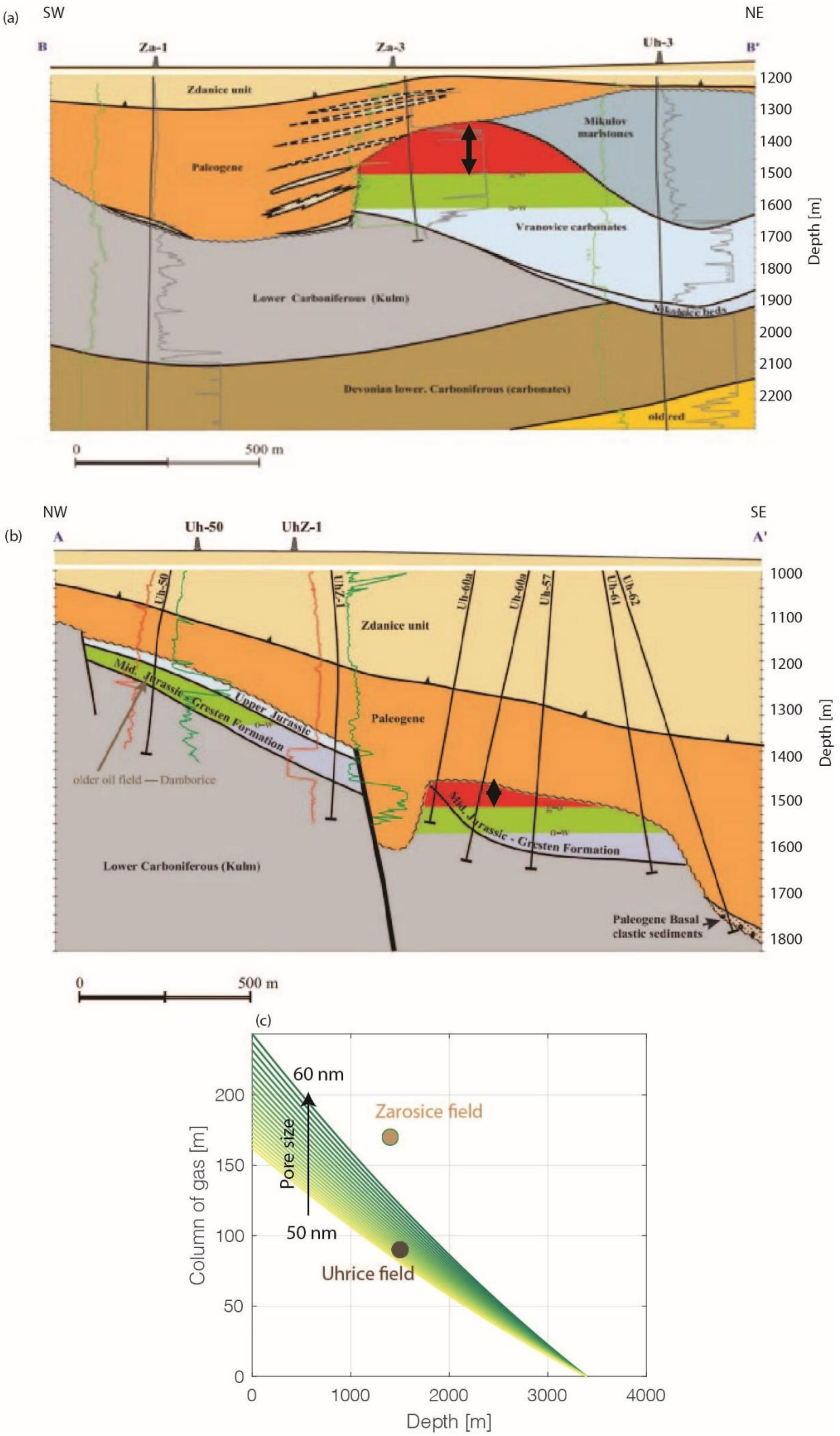
(a)
SW
NE
B
Za-1
Za-3
Uh-3
B'
Zdanice unit
Mikulov marlstones
Paleogene
Vranovice carbonates
Lower Carboniferous (Kulm)
Nikolčice beds
Devonian lower. Carboniferous (carbonates)
old red
1200
1300
1400
1500
1600
1700
1800
1900
2000
2100
2200
Depth [m]
0
500 m
(b)
NW
SE
A
Uh-50
UhZ-1
A'
Uh-60a
Uh-57
Uh-61
Uh-62
Zdanice unit
Paleogene
Mid. Jurassic - Gresten Formation
Upper Jurassic
older oil field — Damborice
Lower Carboniferous (Kulm)
Paleogene Basal clastic sediments
1000
1100
1200
1300
1400
1500
1600
1700
1800
Depth [m]
0
500 m
(c)
60 nm
Pore size
50 nm
Zarosice field
Uhrice field
Column of gas [m]
Depth [m]
0
50
100
150
200
0
1000
2000
3000
4000

Figure S7. Structural trapping of methane differs from hydrogen. Cross-section of a) Uhrice and b) Zarosice fields showing the column height of CH_4 with bold black arrow (Modified after[17]). c) Possible H_2 column height with depth for different pore sizes plotted with the column height of CH_4 in two Czech fields. The CH_4 column height of Zarosice is higher than the column height for structural trapping of H_2, whereas the column height of Uhrice is similar to the column height for structural trapping of H_2.

References

1. DNV GL, June 2020. European Carbon Neutrality: The Importance of as Gas Available: https://eurogas.org/website/wp-content/uploads/2020/06/DNV-GLEurogasReport-Reaching-European-Carbon-Neutrality-Full-Report.pdf
2. Hydrogen Roadmap Europe: a sustainable pathway for the European energy transition, 2019. Available: https://data.europa.eu/doi/10.2843/341510
3. Gas for Climate et al., European Hydrogen Backbone -Analyzing future demand, supply, and transport of hydrogen, Guidehouse, 2021. Available: https://gasforclimate2050.eu/wp content/uploads/2021/06/EHB_Analysing-the-futuredemand-supply-and-transport-of-hydrogen_June 2021_v3.pdf
4. INPEF, SINTEF, and Deloitte finance, 2021. Hydrogen4EU - Charting pathways to enable net zero.Available: https://www.hydrogen4eu.com/
5. Bazzanella, A., and Ausfelder, F., 2017. Low carbon energy and feedstock for the European chemical industry: Technology Study. DECHEMA, Gesellschaft für Chemische Technik und Biotechnologie eV.
6. Impact Assessment Accompanying the proposal for a Regulation of the European Parliament and of the Council on the use of renewable and low-carbon fuels in maritime transport, Jul. 2021.
7. European Commission, Stepping up Europe's 2030 climate ambition - Impact Assessment, Sep. 2020.
8. Tsiropoulos, I., Nijs, W., Tarvydas, D., Ruiz, P., Europäische Kommission, and Gemeinsame Forschungsstelle.,2020. Towards net-zero emissions in the EU energy system by 2050 insights from scenarios in line with the 2030 and 2050 ambitions of the European Green Deal. 2020. Available: https://doi.org/10.2760/081488
9. European Climate Foundation (ECF), Net zero by 2050: from whether to how, ep. 2018. https://europeanclimate.org/wp-content/uploads/2019/12/09-19-net-zero-by-2050-from-whethertohow-executive-summary.pdf.
10. European Commission. Directorate General for Energy., E3 Modelling., Ecofys., and Tractebel 2020, Sectorial integration: long term perspective in the EU energy system. LU: Publications Office, 2020. Available: https://data.europa.eu/doi/10.2833/347937
11. Navigant, Gas for Climate. The optimal role for gas in a net zero emissions energy system,p. 231, Mar. 2019.
12. Öko Institute,2018. The vision scenario for the European Union, 2018. Available: https://www.greensefa.eu/files/doc/docs/9779b987736d6ac6f67f843601efa534.pdf
13. Möst D., Schreiber, S., Herbst, A., Jakob, M.,Martino, A. and Poganietz, W.-R. Eds.,2021. The Future European Energy System: Renewable Energy, Flexibility Options and

Technological Progress. Springer International Publishing, 2021. doi: 10.1007/978-3-030-60914-6
14. Kruck, O., Crotogino, F., Prelicz, R., Rudolph, T., 2013, Assessment of the potential, the actors and relevant business cases for large scale and seasonal storage of renewable electricity by hydrogen underground storage in Europe, HyUnder Report, https://hyunder.eu/wp-content/uploads/2016/01/D3.1_Overview-of-all-known-underground-storage-technologies.pdf
15. Cihlar, J., Mavins, D., van der Leun, K., 2021, Picturing the value of underground gas storage to the European hydrogen system. Guidehouse. https://www.gie.eu/publications/studies/
16. Iglauer, S., 2022. Optimum geological storage depths for structural H2 geo-storage. *Journal of Petroleum Science and Engineering*, *212*, p.109498.
17. Kostelnek, P., Ciprys, V. and Berka, J., 2006. Examples of recently discovered oil and gas fields in the Carpathian foredeep and in the European foreland plate underneath the Carpathian thrust belt, Czech Republic, AAPG Special Volumes#84, p.177-189
